# The graphical method for goodness of fit test in the inverse Weibull distribution based on multiply type-II censored samples 

**DOI:** 10.1186/s40064-015-1554-x

**Published:** 2015-12-12

**Authors:** Suk-Bok Kang, Jun-Tae Han

**Affiliations:** Department of Statistics, Yeungnam University, Gyeongsan, 712-749 Korea; Statistics and Analysis Team, Korea Student Aid Foundation, Seoul, 100-753 Korea

**Keywords:** Approximate maximum likelihood estimator, Goodness-of-fit test, Inverse Weibull distribution, Multiply type-II censored sample

## Abstract

Many studies have considered a truncated and censored samples which are type-I, type-II and hybrid censoring scheme. The inverse Weibull distribution has been utilized for the analysis of life testing and reliability data. Also, this distribution is a very flexible distribution. The inverse Rayleigh distribution and inverse exponential distribution are a special case of the inverse Weibull distribution. In this paper, we derive the approximate maximum likelihood estimators (AMLEs) of the scale parameter and the shape parameter in the inverse Weibull distribution under multiply type-II censoring. We also propose a simple graphical method for goodness-on-fit test based on multiply type-II censored samples using AMLEs.

## Background

The probability density function (PDF) and the cumulative distribution function (CDF) of the two-parameter inverse Weibull distribution are given by

1.1 $$\begin{aligned} g\left( x; \sigma , \lambda \right) ={\lambda \sigma ^{-\lambda }}x^{-(\lambda +1)}\mathrm {exp} \left[ {-( x\sigma )^{-\lambda }}\right] , \quad  x>\mathrm 0,\sigma >0,\lambda >0 \end{aligned}$$

and

1.2 $$\begin{aligned} G\left( x; \sigma , \lambda \right) =\mathrm {exp}\left[ {-( x\sigma )^{-\lambda }}\right] ,\quad ~ x>\mathrm 0,\sigma >0,\lambda >0, \end{aligned}$$

where $\sigma$ and $\lambda$ are scale and shape parameters respectively.

This distribution has been recently proposed as a model in the analysis of life testing data. Many authors have discussed estimation of the parameters and associated inference, for example, Calabria and Pulcini (1990, 1994; Maswadah 2003; Mahmoud et al. 2003).

In life testing and reliability experiments, it is well known that the lifetimes of test units may not be always observed exactly. There are also situations in which the removal of units prior to failure is pre-planned because of the time or cost limitations associated with testing. The type-I and type-II censoring are the most common censoring schemes, but the typical type-I and type-II censoring do not have flexibility. The type-II censoring scheme is a special case of the multiply type-II censoring scheme. Multiply type-II censored sampling arises in a life-testing experiment whenever the experimenter does not record the failure times of some units placed on a life testing.

The approximated maximum likelihood estimating method for the Rayleigh distribution was first developed by Balakrishnan (1989). Fei et al. (1995) studied the estimation for the two-parameter Weibull distribution and extreme-value distribution under multiply type-II censoring. They compared the mean squared errors of the maximum likelihood estimators, approximate maximum likelihood estimators (AMLEs), and best linear unbiased estimators (BLUEs) of the parameters in the extreme value distribution.

Goodness-of-fit tests were discussed by several authors. Porter III et al. (1992) developed three modified Kolmogorov-Smirnov, Anderson-Darling, and Cramer-von Mises tests for the Pareto distribution based on the complete samples. Shimokawa and Liao (1999) studied the goodness of fit test for the extreme value and Weibull distribution, when the population parameters are estimated from a complete sample by graphical plotting techniques. Puig and Stephens (2000) studied some tests of fit for the Laplace distribution based on the empirical distribution function (EDF) statistics and the application of the Laplace distribution in the least absolute deviations regression. In addition, Choulakian and Stephens (2001) discussed estimation of parameters and goodness-of-fit tests for the generalized Pareto distribution.

The objective of the our study is to derive the AMLEs of the scale parameter $\sigma$ and the shape parameter $\lambda$ based on multiply type-II censored samples. We also propose a simple graphical method for goodness-of-fit test based on multiply type-II censored samples using AMLEs.

The paper is organized as follows. “Approximate maximum likelihood estimators” describes estimation of the scale and shape parameter under multiply type-II censored samples. “Graphical methods in the goodness-of-t tests” describes graphical methods in the goodness-of-fit tests. In “Illustrative examples”, we apply graphical method using two example data set. Finally, “Conclusions” concludes the paper and gives some recommendations for future work.

## Approximate maximum likelihood estimators

We assume that *n* items are put on a life test, but only $a_1$th, $a_2$th, …, $a_s$th failures are observed, the rest are unobserved or missing, where $a_1$, $a_2$,…, $a_s$ are considered to be fixed.

If *X* is an inverse Weibull random variable, then $Y=logX$ has extreme-value distribution with location $\mu = log(1/\sigma )$ and scale parameter $\theta = 1/\lambda$ with PDF and CDF given respectively as;

2.1 $$\begin{aligned} f\left( y; \mu , \theta \right) ={1\over {\theta }} \mathrm{exp} \left( -{{y-\mu }\over {\theta }} \right) \mathrm{exp} \left[ -\mathrm{exp} \left( -{{y-\mu }\over {\theta }} \right) \right] \end{aligned}$$

and

2.2 $$\begin{aligned} F\left( y; \mu , \theta \right) =\mathrm{exp} \left[ -\mathrm{exp} \left( -{{y-\mu }\over {\theta }} \right) \right] . \end{aligned}$$

Let us assume that the following multiply type-II censored sample from a sample of size *n* is $y_{a_1 : n} \le y_{a_2 : n} \le \cdots \le y_{a_s : n}$, where $1~\le ~a_1~<~a_2~<~\cdots ~<~a_s~\le n$, $a_0 = 0$, $a_{s+1} = n+1$, $F(y_{a_0 :n} )=0$, and $F(y_{a_{s+1} :n} )=1$.

The likelihood function based on the multiply type-II censored sample is given by

2.3 $$\begin{aligned} L&= {1\over {\sigma ^s}} {n!\over \prod _{j=1}^{s+1} (a_j - a_{j-1} -1 )! } [F(z_{a_1 :n} ) ]^{a_1 - 1} [1 - F(z_{a_s :n} )]^{n-a_s }\\&\quad \quad \times \prod _{j=1}^s f(z_{a_j : n} ) \left[ \prod _{j=2}^s [F(z_{a_j : n} )- F(z_{a_{j-1} :n} )]^{a_j - a_{j-1} -1} \right] , \nonumber \end{aligned}$$

where $z_{i : n} = ({y_{i : n} - \mu }) / \theta$, and *f*(*z*) and *F*(*z*) are the pdf and the cdf of the standard extreme-value distribution, respectively.

Since ${f'(z) / f(z)}=e^{-z}-1$, we can obtain the likelihood equations as follows;

2.4 $$\begin{aligned} {\partial \mathrm{ln}L \over \partial \theta }&= - {1 \over \theta } \Biggl [ s+(a_1 -1) e^{-z_{a_1 :n}} z_{a_1 :n} - (n-a_s ) {f(z_{a_s :n}) \over 1-F(z_{a_s :n})}z_{a_s :n} +\sum _{j=1}^s e^{-z_{a_j :n}}z_{a_j :n} \nonumber \\&\quad - \sum _{j=1}^s z_{a_j :n} + \sum _{j=2}^s (a_j - a_{j-1} -1) {f(z_{a_j :n})z_{a_j :n} - f(z_{a_{j-1} :n}) z_{a_{j-1} :n} \over F(z_{a_j :n} )-F(z_{a_{j-1} :n})} \Biggr ] \\&=0, \nonumber \end{aligned}$$

and

2.5 $$\begin{aligned} {\partial \mathrm{ln}L \over \partial \mu }&= - {1 \over \theta } \Biggl [ (a_1 -1) e^{-z_{a_1 :n}}- (n-a_s ) {f(z_{a_s :n}) \over 1-F(z_{a_s :n})} +\sum _{j=1}^s e^{-z_{a_j :n}} - s\nonumber \\&\quad + \sum _{j=2}^s (a_j - a_{j-1} -1) {f(z_{a_j :n}) - f(z_{a_{j-1} :n}) \over F(z_{a_j :n} )-F(z_{a_{j-1} :n})} \Biggr ] \\&= 0.\nonumber \end{aligned}$$

Since the likelihood equations are very complicated, the equations (2.4) and (2.5) do not admit explicit solutions for $\theta$ and $\mu$, respectively.

Let $\xi _i = F^{-1}(p_i)=-\mathrm{ln}\left[ -\mathrm{ln}p_i\right]$ where $p_i = { i / (n+1)}$, $q_i = 1-p_i$. Further, we may expand the following function in a Taylor series around the points $\xi _{a_i}$ and ($\xi _{a_{i-1}}$, $\xi _{a_i}$) respectively.

We can approximate the following functions by

2.6 $$\begin{aligned} {f(z_{a_s :n}) \over 1-F(z_{a_s :n})}\simeq & \,\kappa _1 +\delta _1 z_{a_s :n},\end{aligned}$$

2.7 $$\begin{aligned} e^{-z_{a_j :n}}\simeq & \, e^{-\xi _{a_j}}\left( 1+\xi ^2_{a_j}\right) - e^{-\xi _{a_j}} z_{a_j :n},\end{aligned}$$

2.8 $$\begin{aligned} {f(z_{a_j :n}) \over F(z_{a_j :n} )-F(z_{a_{j-1} :n})}\simeq & \, \alpha _{1j} +\beta _{1j} z_{a_j :n}+\gamma _{1j} z_{a_{j-1} :n}, \end{aligned}$$

and

2.9 $$\begin{aligned} {f(z_{a_{j-1} :n}) \over F(z_{a_j :n} )-F(z_{a_{j-1} :n})} \simeq \alpha _{2j} +\beta _{2j} z_{a_j :n}+\gamma _{2j} z_{a_{j-1} :n}, \end{aligned}$$

where

$$\begin{aligned} \kappa _1&= {1 \over q_{a_s}} \left[ f(\xi _{a_s})- \xi _{a_s}f'(\xi _{a_s}) -{f^2(\xi _{a_s}) \over q_{a_s}}\xi _{a_s} \right] , \delta _1 = {1 \over q_{a_s}} \left[ f'(\xi _{a_s})+ {f^2(\xi _{a_s}) \over q_{a_s}} \right] ,\\ \alpha _{1j}&= {\left[ (1+K_j)f(\xi _{a_j}) - \xi _{a_j}f'(\xi _{a_j}) \right] \over p_{a_j} - p_{a_{j-1}}},~~ \beta _{1j} = {f'(\xi _{a_j}) \over p_{a_j} - p_{a_{j-1}}} - \left[ {f(\xi _{a_j}) \over p_{a_j} - p_{a_{j-1}}} \right] ^2 ,\\ \gamma _{1j}&= {f(\xi _{a_j})f(\xi _{a_{j-1}})\over \left[ p_{a_j} - p_{a_{j-1}}\right] ^2},~~ \alpha _{2j} = {\left[ (1+K_j)f(\xi _{a_{j-1}}) - \xi _{a_{j-1}}f'(\xi _{a_{j-1}}) \right] \over p_{a_j} - p_{a_{j-1}}},\\ \beta _{2j}&= -\gamma _{1j} ,~~~ \gamma _{2j} = {f'(\xi _{a_{j-1}}) \over p_{a_j} - p_{a_{j-1}}} + \left[ {f(\xi _{a_{j-1}}) \over p_{a_j}-p_{a_{j-1}}} \right] ^2, \\ K_j&= {f(\xi _{a_j})\xi _{a_j} - f(\xi _{a_{j-1}})\xi _{a_{j-1}} \over p_{a_j} - p_{a_{j-1}}}. \end{aligned}$$

By substituting the equations (2.6)−(2.9) into the equation (2.4), we can derive an estimator of $\theta$ as follows;

2.10 $$\begin{aligned} {\hat{\theta }} = {-B_1+\sqrt{{B_1}^2 -4s C_1} \over 2s}, \end{aligned}$$

where

$$\begin{aligned} B_1&= (a_1 -1)e^{-\xi _{a_1}}\left( 1+\xi ^2_{a_1}\right) y_{a_1 :n} -(n-a_s )\kappa _1 y_{a_s :n} + \sum _{j=1}^s e^{-\xi _{a_j}}(1+\xi ^2_{a_j})y_{a_j :n} - \sum _{j=1}^s y_{a_j :n} \\&\quad + \sum _{j=2}^s (a_j - a_{j-1} -1)(\alpha _{1j}y_{a_j :n} - \gamma _{2j}y_{a_{j-1} :n})- \Biggl [(a_1 -1) e^{-\xi _{a_j} }\xi ^2_{a_j} -(n-a_s )\kappa _1\\&\quad + \sum _{j=1}^s e^{-\xi _{a_j}}\left( 1+\xi ^2_{a_j}\right) - s + \sum _{j=2}^s (a_j - a_{j-1}-1)(\alpha _{1j} - \gamma _{2j})\Biggl ] {\hat{\mu }}, \\ C_1&= -(a_1 -1)e^{-\xi _{a_1}} (y_{a_1 :n}-{\hat{\mu }})^2 - (n-a_s )\delta _1(y_{a_s :n}-{\hat{\mu }})^2 - \sum _{j=1}^s e^{-\xi _{a_j}}(y_{a_j :n}-{\hat{\mu }})^2\\&\quad +\sum _{j=2}^s (a_j - a_{j-1}-1) \biggl [\beta _{1j}(y_{a_j :n}-{\hat{\mu }})^2+2 \gamma _{1j}(y_{a_j :n}-{\hat{\mu }})(y_{a_{j-1} :n}-{\hat{\mu }})\\&\quad -\gamma _{2j}(y_{a_{j-1} :n}-{\hat{\mu }})^2 \biggl ]. \end{aligned}$$

Next, equation (2.5) does not admit an explicit solution for $\mu$. But we can expand the following function as follows;

2.11 $$\begin{aligned} {f(z_{a_j :n})-f(z_{a_{j-1} :n}) \over F(z_{a_j :n} )-F(z_{a_{j-1} :n})} \simeq \alpha _{3j} +\beta _{3j} z_{a_j :n}+\gamma _{3j} z_{a_{j-1} :n} \end{aligned}$$

where $$\alpha _{3j}=\alpha _{1j}-\alpha _{2j}$$, $$\beta _{3j}=\beta _{1j}-\beta _{2j}$$, and $$\gamma _{3j}=\gamma _{1j}-\gamma _{2j}$$.

By substituting the equations (2.6), (2.7), and (2.11) into the equation (2.5), we can derive an estimator of $\mu$ as follows;

2.12 $$\begin{aligned} {\hat{\mu }}={E \over D}, \end{aligned}$$

where

$$\begin{aligned} D&= A_\mu C_2 - A_2 C_\mu ,~~E=A_\mu B_2 - A_2 B_\mu , \\ A_\mu&= (a_1 -1)e^{-\xi _{a_1}}(1+\xi ^2_{a_1}) - (n-a_s )\kappa _1 + \sum _{j=1}^s e^{-\xi _{a_j}}(1+\xi ^2_{a_j}) -s + \sum _{j=2}^s (a_j - a_{j-1} -1)\alpha _{3j},\\ B_\mu&= -(a_1 -1)e^{-\xi _{a_1}} y_{a_1 :n} - (n-a_s )\delta _1 y_{a_s :n} - \sum _{j=1}^s e^{-\xi _{a_j}}y_{a_j :n} \\&\quad + \sum _{j=2}^s (a_j - a_{j-1} -1)(\beta _{3j}y_{a_j :n} + \gamma _{3j}y_{a_{j-1} :n}),\\ C_\mu&= -(a_1 -1)e^{-\xi _{a_1}}- (n-a_s )\delta _1 - \sum _{j=1}^s e^{-\xi _{a_j}} + \sum _{j=2}^s (a_j - a_{j-1} -1)(\beta _{3j}+ \gamma _{3j}),\\ A_2&= s+ (a_1 -1)e^{-\xi _{a_1}}\xi ^2_{a_1} -(n-a_s )\kappa _2 + \sum _{j=1}^s e^{-\xi _{a_j}}\xi ^2_{a_j} + \sum _{j=2}^s (a_j - a_{j-1} -1) \alpha _{4j}, \\ B_2&= (a_1 -1)e^{-\xi _{a_1}}(1-\xi _{a_1}) y_{a_1 :n} -(n-a_s )\delta _2 y_{a_s :n} + \sum _{j=1}^s e^{-\xi _{a_j}}(1-\xi _{a_j})y_{a_j :n} \\&\quad + \sum _{j=1}^s y_{a_j :n} + \sum _{j=2}^s (a_j - a_{j-1} -1)(\beta _{4j}y_{a_j :n} + \gamma _{4j}y_{a_{j-1} :n}),\\ C_2&= (a_1 -1)e^{-\xi _{a_j}}(1-\xi _{a_j}) -(n-a_s )\delta _2 + \sum _{j=1}^s e^{-\xi _{a_j}}(1-\xi _{a_j}) -s \\&\quad + \sum _{j=2}^s (a_j - a_{j-1} -1)(\beta _{4j} + \gamma _{4j}),\\ \kappa _2&= -{\xi ^2_{a_s} \over q_{a_s}} \left[ f'(\xi _{a_s})+{f^2(\xi _{a_s}) \over q_{a_s}} \right] ,\quad\delta _2 = {1 \over q_{a_s}} \left[ f(\xi _{a_s})+ \xi _{a_s}f'(\xi _{a_s}) +{f^2(\xi _{a_s}) \over q_{a_s}}\xi _{a_s} \right] ,\\ \alpha _{4j}&= K_j^2 - { \xi ^2_{a_j} f'(\xi _{a_j}) - \xi ^2_{a_{j-1}} f'(\xi _{a_{j-1}}) \over p_{a_j} - p_{a_{j-1}}},\quad\beta _{4j} = {\left[ (1-K_j)f(\xi _{a_j}) + \xi _{a_j}f'(\xi _{a_j}) \right] \over p_{a_j} - p_{a_{j-1}}} ,\\ \gamma _{4j}&= -{\left[ (1-K_j)f(\xi _{a_{j-1}}) + \xi _{a_{j-1}}f'(\xi _{a_{j-1}}) \right] \over p_{a_j} - p_{a_{j-1}}}. \end{aligned}$$

Since $\theta = 1 / \lambda$ and $\mu = log(1/\sigma )$, we can obtain the AMLEs of the shape parameter $\lambda$ and the scale parameter $\sigma$ as follows; ${\hat{\lambda }}= 1 / {\hat{\theta }}$ and ${\hat{\sigma }} = 1 / e^{\hat{\mu }}$.

## Graphical methods in the goodness-of-fit tests

In this section, we consider a graphical method for goodness on fit test in the inverse Weibull distribution based on multiply type-II censored samples using AMLEs.

### Modified normalized sample Lorenz curve

The Lorenz curve is extensively used in the study of income distribution and used to be a powerful tool for the analysis of a variety of scientific problems.

Cho et al. (1999) proposed the transformed Lorenz curve that can be used in the study of symmetric distribution. The transformed Lorenz curve is defined by

3.1 $$\begin{aligned} TL(r_{i})={\sum _{j=1}^{i} X_{j:n}\over \sum _{j=1}^{n}X_{j:n}}, ~~r_i={i \over n}, ~~i=1,2,\ldots ,n. \end{aligned}$$

Kang and Cho (2001) proposed the normalized sample Lorenz curve (NSLC) for the complete sample as follows;

3.2 $$\begin{aligned} NSLC(r_i)={TSL(r_i)\over TSL_F(r_i)}, ~~r_i={i\over n}, ~~i=1,2,\ldots ,n, \end{aligned}$$

where

$$\begin{aligned} TSL(r_i) & =  {\sum _{j=1}^{i}(X_{j:n}-X_{1:n})\over \sum _{j=1}^{n}(X_{j:n}-X_{1:n})}-r_i+1,\\ TSL_F(r_i) & = {\sum _{j=1}^{i}\left[ F^{-1}(p_{j})-F^{-1}(p_1)\right] \over \sum _{j=1}^{n}\left[ F^{-1}(p_j)-F^{-1}(p_1)\right] }-r_i+1. \end{aligned}$$

Now, we propose modified NSLC based on multiply type-II censored samples.

The modified NSLC based on multiply type-II censored samples is given by

3.3 $$\begin{aligned} MNSLC(r_{i})={MTSL(r_i)\over MTSL_F(r_i)},~~~ r_i={a_i \over n}, ~~i=1,2,\ldots ,s, \end{aligned}$$

where

$$\begin{aligned} MTSL(r_{i})= & {} {\sum _{j=1}^{i}(X_{a_j:n}-X_{a_1:n}) \over \sum _{j=1}^{s}(X_{a_j:n}-X_{a_1:n})} - r_i + 1, \\ MTSL_F(r_i)= & {} { \sum _{j=1}^i \left[ F^{-1}(p_{a_j};{\hat{\sigma }},{\hat{\lambda }})-F^{-1}(p_{a_1};{\hat{\sigma }},{\hat{\lambda }}) \right] \over \sum _{j=1}^s \left[ F^{-1}(p_{a_j};{\hat{\sigma }},{\hat{\lambda }})-F^{-1}(p_{a_1};{\hat{\sigma }},{\hat{\lambda }}) \right] } -r_i +1. \end{aligned}$$

**Fig. 1:**
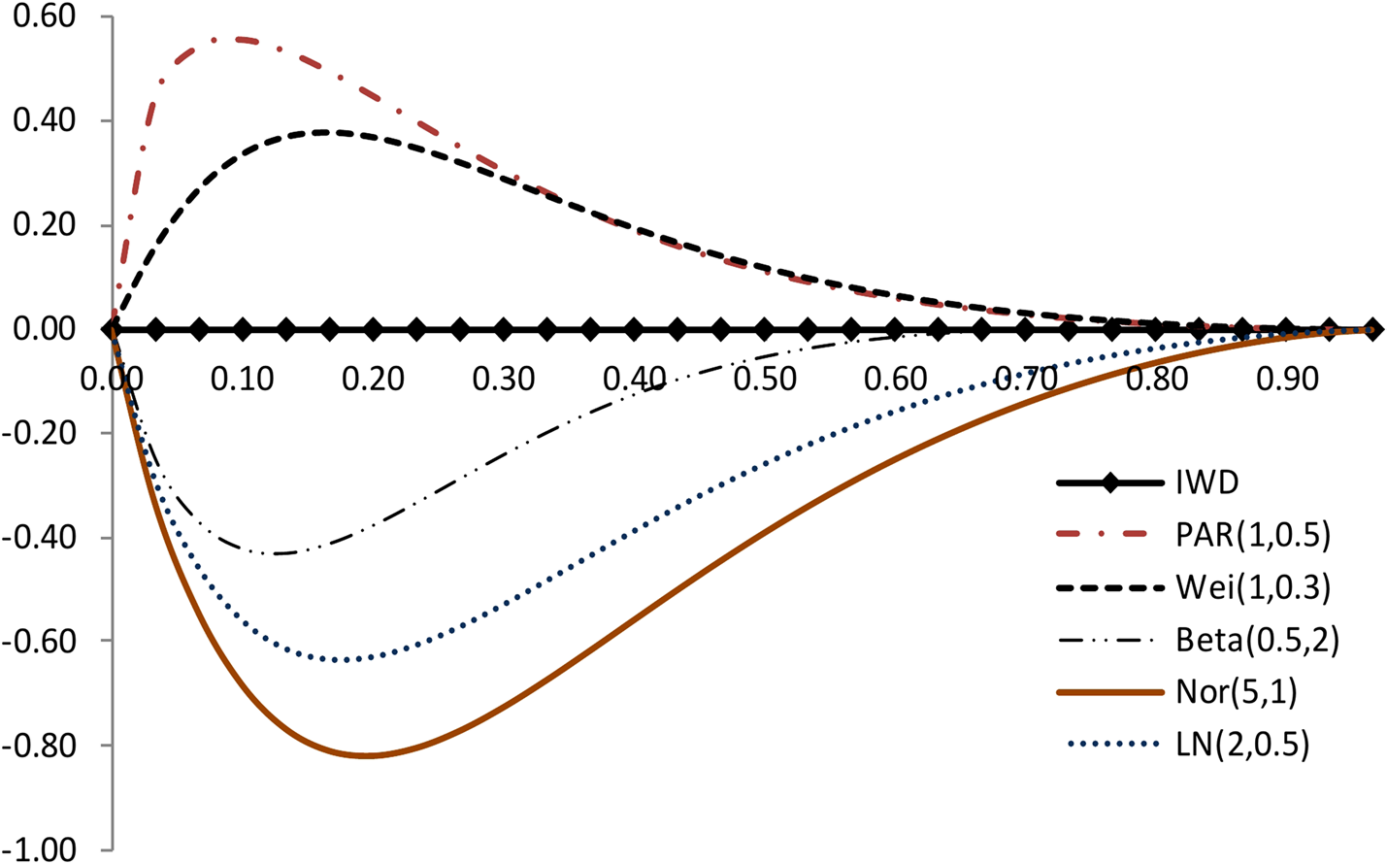
Modified NSLC plot: complete data (*n* = 30)

**Fig. 2:**
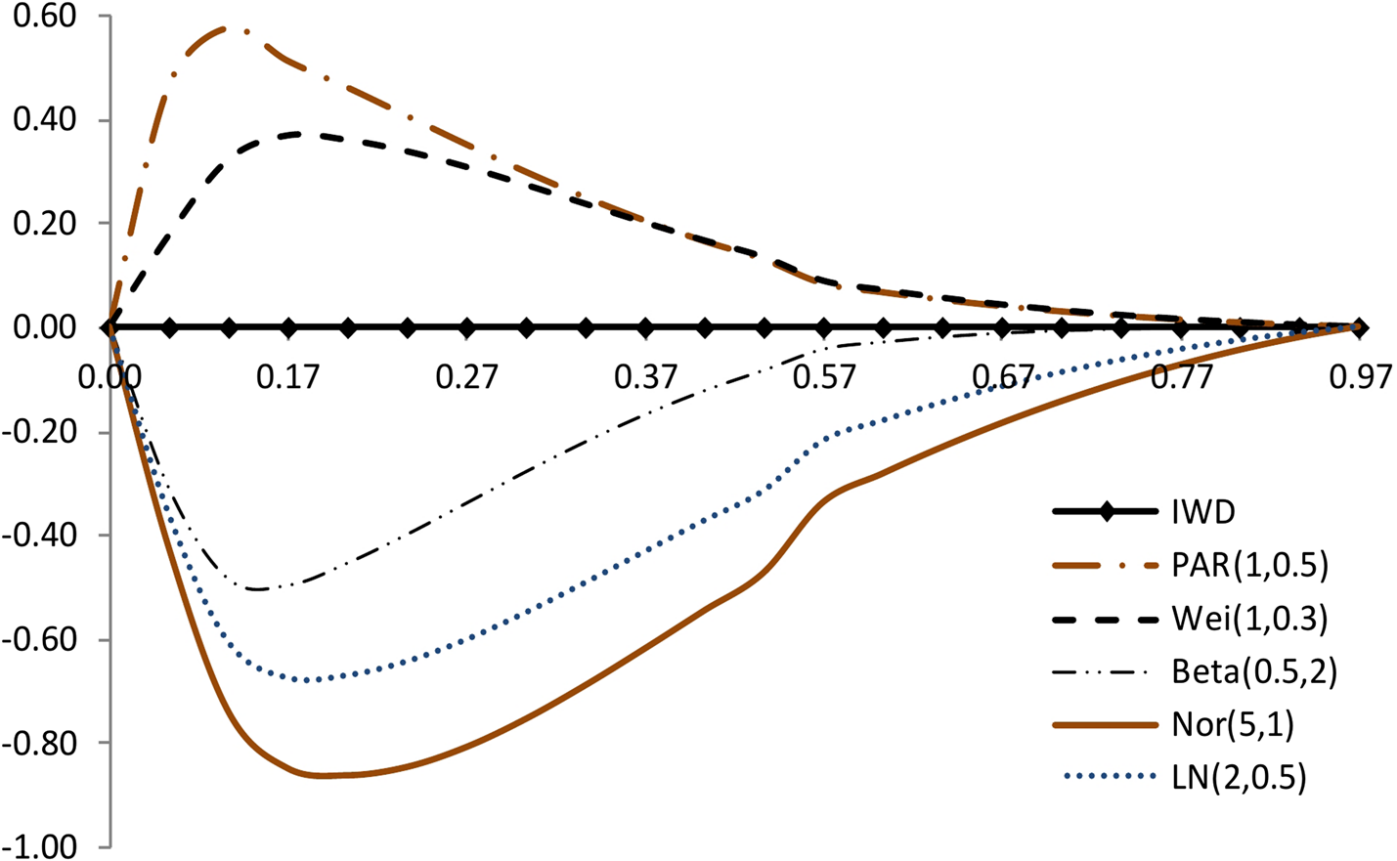
Modified NSLC plot: multiply type-II censored data (*n* = 30, $a_j$ = 1, 5–13, 17–25, 28–30)

Also, we propose the modified NSLC plot for multiply type-II censored samples using (X,Y) = $(1-r_i, 1-MNSLC_i)$. If data come from the inverse Weibull distribution, the modified NSLC plot is $y=0$ (see, Figs. 1, 2). The value of $1-MNSLC_i$ increases and then decreases as $1-r_i$ increases when the alternative is Pareto and Weibull distributions. But the value of $1-MNSLC_i$ decreases and then increases as $1-r_i$ increases when the alternative is beta, lognormal and normal distributions.

### Test based on spacing of EDF

We have an idea for plot and test statistics based on the spacing of the EDF.

$F(x_{a_i:n})$ has a different spacing of order statistics at all the distribution. We use the range $F(x_{a_i:n})-F(x_{a_1:n})$ between $a_i$th point and the $a_1$th point. So we propose the plot for multiply Type-II censored samples by

3.4 $$\begin{aligned} (x,~y)=\left( {a_i \over n+1 },~ { R_{i} \over P_i}-1 \right) ,~~i=1,2,..,s, \end{aligned}$$

where

3.5 $$\begin{aligned} R_{i}= & {} { \sum _{j=1}^i F(x_{a_j:n},{\hat{\sigma }},{\hat{\lambda }}) - F(x_{a_1:n},{\hat{\sigma }},{\hat{\lambda }}) \over F(x_{a_s:n},{\hat{\sigma }},{\hat{\lambda }}) - F(x_{a_1:n},{\hat{\sigma }},{\hat{\lambda }}) } +1, \end{aligned}$$

3.6 $$\begin{aligned} P_i= & {} { \sum _{j=1}^i a_{j:n} - a_{1:n} \over a_{s:n} - a_{1:n} } +1. \end{aligned}$$

**Fig. 3:**
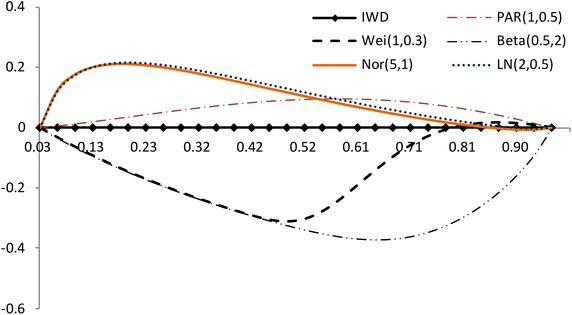
Plot based on the spacing of the EDF: complete data (*n* = 30)

**Fig. 4:**
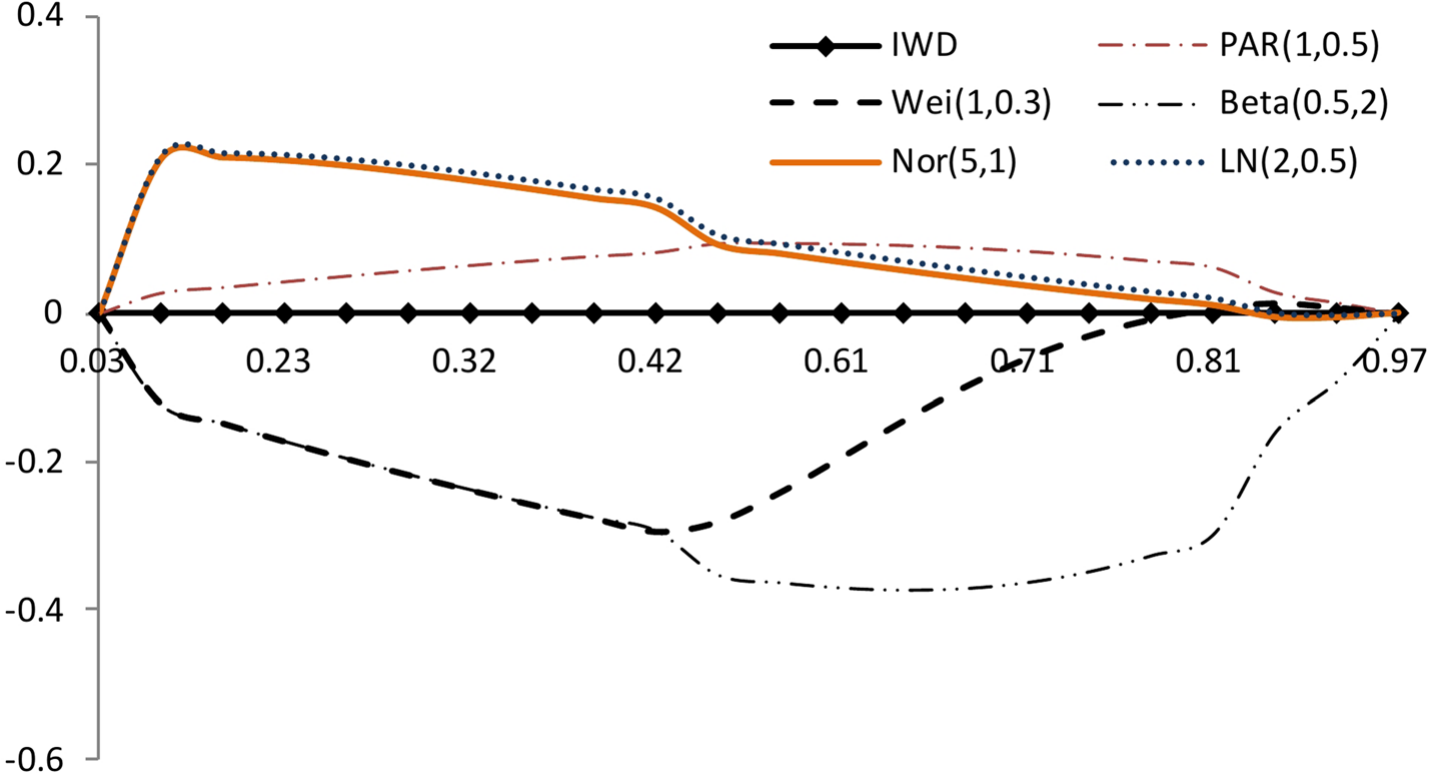
Plot based on the spacing of the EDF: multiply type-II censored data (*n* = 30, $a_j$ = 1, 5–13, 17–25, 28–30)

If data come from inverse Weibull distribution, the above plot is $y=0$ (see, Figs. 3, 4).

The value of $(R_{i} / P_i)-1$ increases and then decreases as ${a_i / (n+1) }$ increases when the alternative is normal and lognormal distributions. But the value of $(R_{i} / P_i)-1$ decreases and then increases as ${a_i / (n+1) }$ increases when the alternative is Weibull and beta distributions. The normal alternative distribution and the lognormal alternative distribution are similar.

## Illustrative examples

In this section, we show some illustrative examples using real data sets and discuss the results of examples.

### Example 1: the ball bearings in the life test

The data given here arose in tests on the endurance of deep groove ball bearings. They were originally discussed by Lieblein and Zelen (1956), who assumed that the data came from a Weibull distribution. The data are the number of million revolutions before failure for each of the 23 ball bearings in the life test:

17.88, 28.92, 33.00, 41.52, 42.12, 45.60, 48.40, 51.84, 51.96, 54.12, 55.56, 67.80, 68.64, 68.64, 68.88, 84.12, 93.12, 98.64, 105.12, 105.84, 127.92, 128.04, 173.40.

To work with the inverse Weibull distribution, the 23 failure times are converted to inverse failure times:

0.006, 0.008, 0.008, 0.009, 0.010, 0.010, 0.011, 0.012, 0.015, 0.015, 0.015, 0.015, 0.018, 0.018, 0.019, 0.019, 0.021, 0.022, 0.024, 0.024, 0.030, 0.035, 0.056.

For complete data, we can obtain the AMLEs ${\hat{\lambda }}=2.121929$ and ${\hat{\sigma }}=81.450162$. For this example of $n=23$, $s=16$($a_j=1, 2, 5{-}14, 18{-}21$), and the multiply Type-II censored samples are 0.006, 0.008, –, –, 0.010, 0.010, 0.011, 0.012, 0.015, 0.015, 0.015, 0.015, 0.018, 0.018, –, –, –, 0.022, 0.024, 0.024, 0.030, –, –, we can obtain the AMLEs ${\hat{\lambda }}=2.062999$ and ${\hat{\sigma }}=80.986041$.

**Fig. 5:**
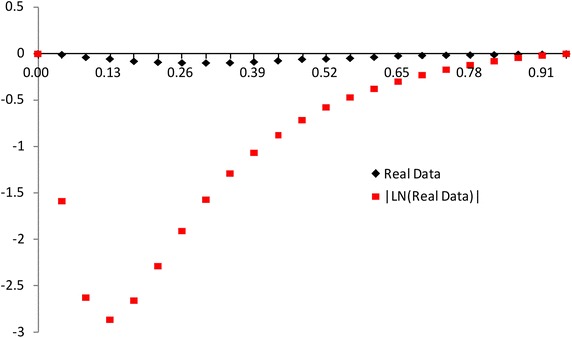
Modified NSLC plot [Example 1: complete data (*n* = 30)]

**Fig. 6:**
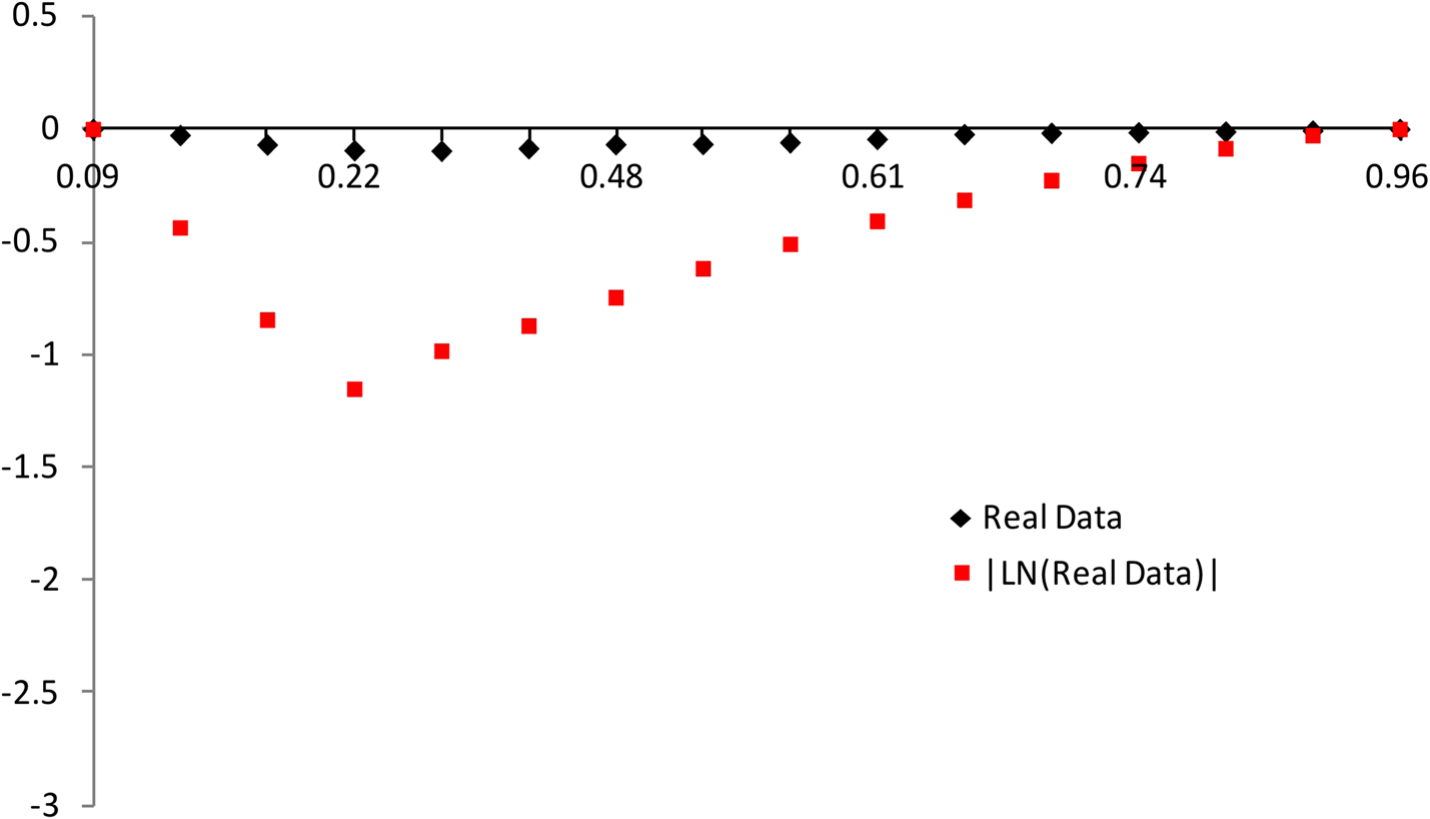
Modified NSLC plot [example 1: multiply type-II censored data (*n* = 30, $a_j$ = 1, 5–13, 17–25, 28–30)]

**Fig. 7:**
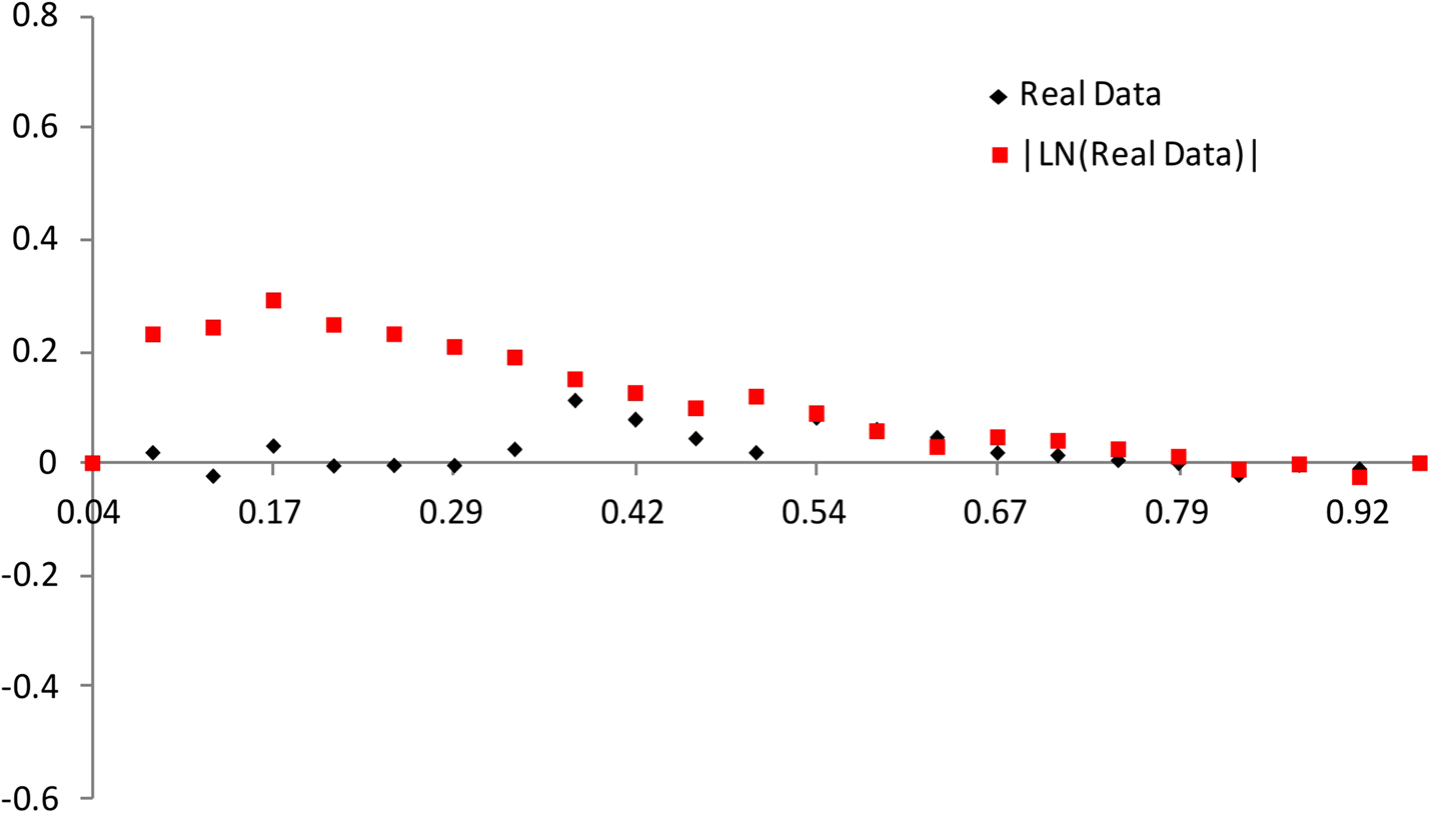
Plot based on the spacing of the EDF [example 1: complete data (*n* = 30)]

**Fig. 8:**
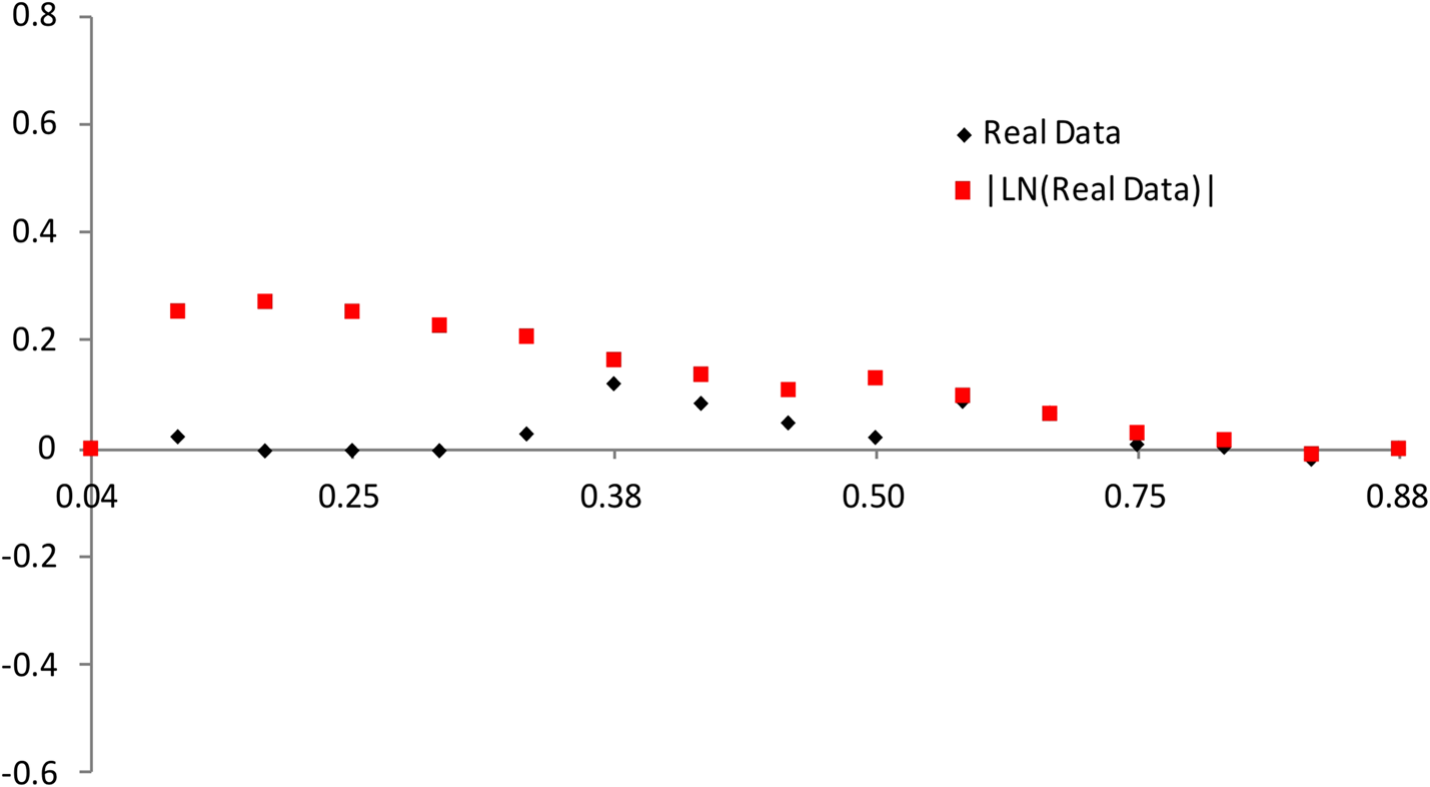
Plot based on the spacing of the EDF (example 1: multiply type-II censored data [*n* = 30, $a_j$  = 1, 5–13, 17–25, 28–30)]

We can picture the proposed plots for multiply Type-II censored samples using the AMLEs ${\hat{\lambda }}$ and ${\hat{\sigma }}$ (see Figs. 5, 6, 7, 8). It is easy to see that the modified NSLC plot has good performance for complete data or multiply Type-II censored samples. The modified NSLC plot is more sensitive than the plot based on spacing of EDF.

### Example 2: maximum flood levels of the susquehenna river

Data given by Dumonceaux and Antle (1963), represents the maximum flood levels (in million of cubic feet per second) of the Susquehenna River at Harrisburg, Pennsylvenia over 20 four-year periods (1890–1969) as follows;

0.654, 0.613, 0.315, 0.449, 0.297, 0.402, 0.379, 0.423, 0.379, 0.324, 0.269, 0.740, 0.418, 0.412, 0.494, 0.416, 0.338, 0.392, 0.484, 0.265.

This data had been utilized earlier by Maswadah (2003). He showed a rough indication of the goodness-of-fit for the model, due to the smallness of the sample seize, by plotting the empirical CDF and the CDF of the inverse Weibull distribution using the maximum likelihood estimators of the parameters. Maswadah (2003) showed that the inverse Weibull distribution provides a good fit to these data, which demonstrated the usefulness of the inverse Weibull distribution in modeling extreme value data, as well as its applicability in the analysis of natural phenomena (flood, drought, rainfall, etc.).

We now apply the proposed estimators to these data, and assess their goodness of-fit. For complete data, we can obtain the AMLEs ${\hat{\lambda }}=4.335915$ and ${\hat{\sigma }}=2.783092$. For this example of $n=20$, $s=15$($a_j=1{-} 7, 11{-} 18$), and the multiply Type-II censored samples are 0.265, 0.269, 0.297, 0.315, 0.324, 0.338, 0.379, –, –, –, 0.412, 0.416, 0.418, 0.423, 0.449, 0.484, 0.494, 0.613, –, –, we can obtain the AMLEs ${\hat{\lambda }}=4.132622$, and ${\hat{\sigma }}=2.770161$.

**Fig. 9:**
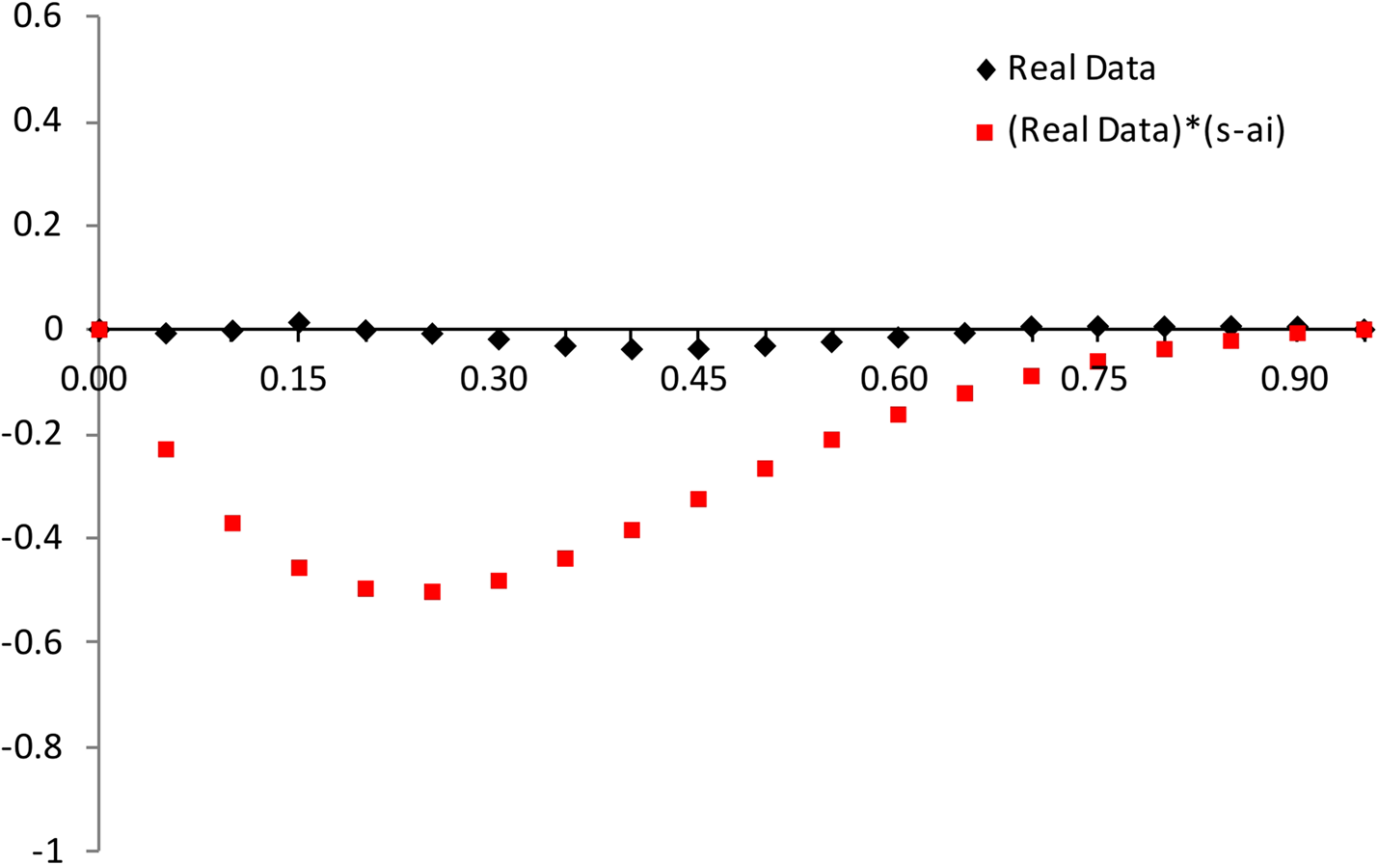
Modified NSLC plot (example 2: complete data (*n* = 30))

**Fig. 10:**
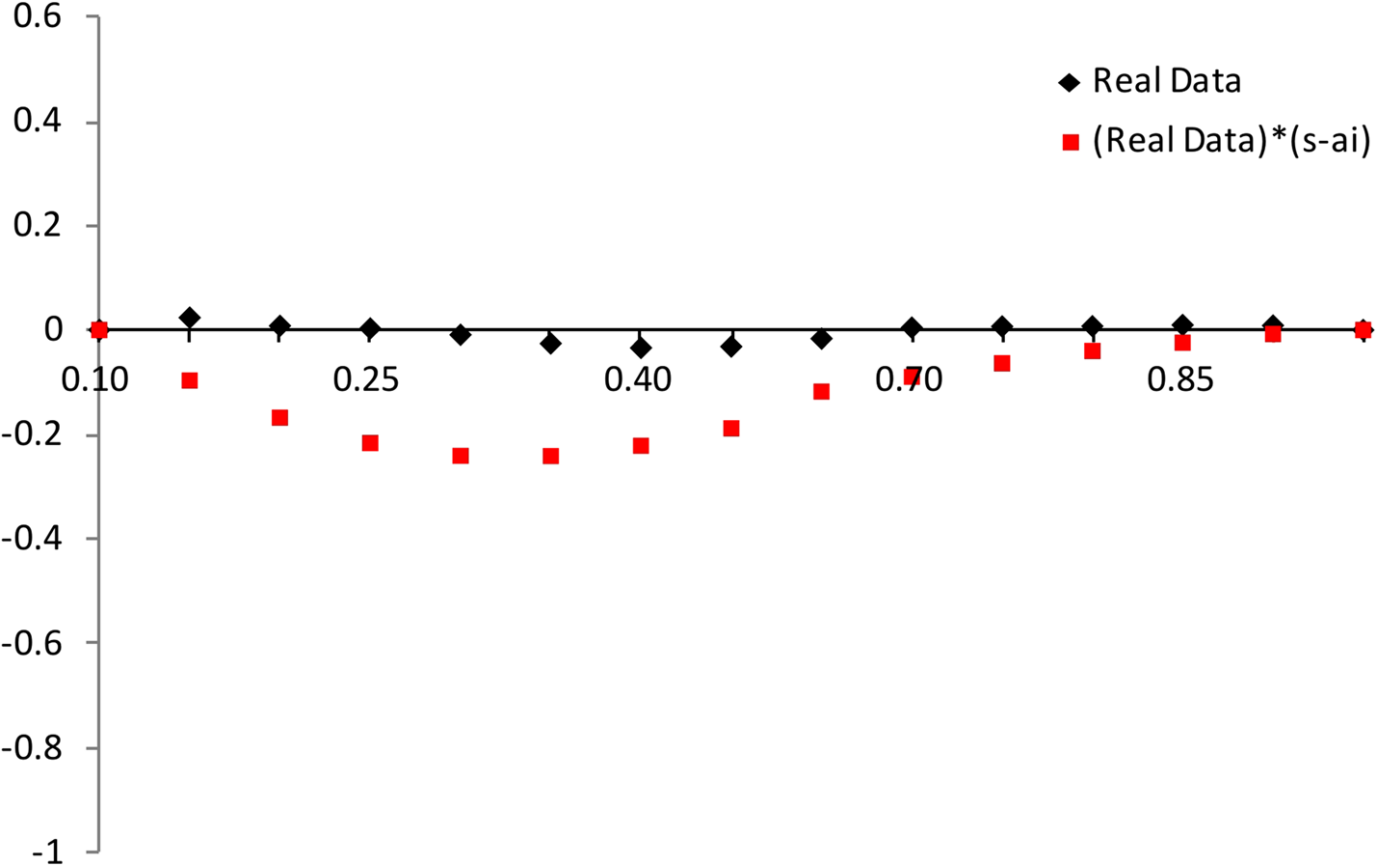
Modified NSLC plot [example 2: multiply type-II censored data (*n* = 30, $a_j$ = 1, 5–13, 17–25, 28–30)]

**Fig. 11:**
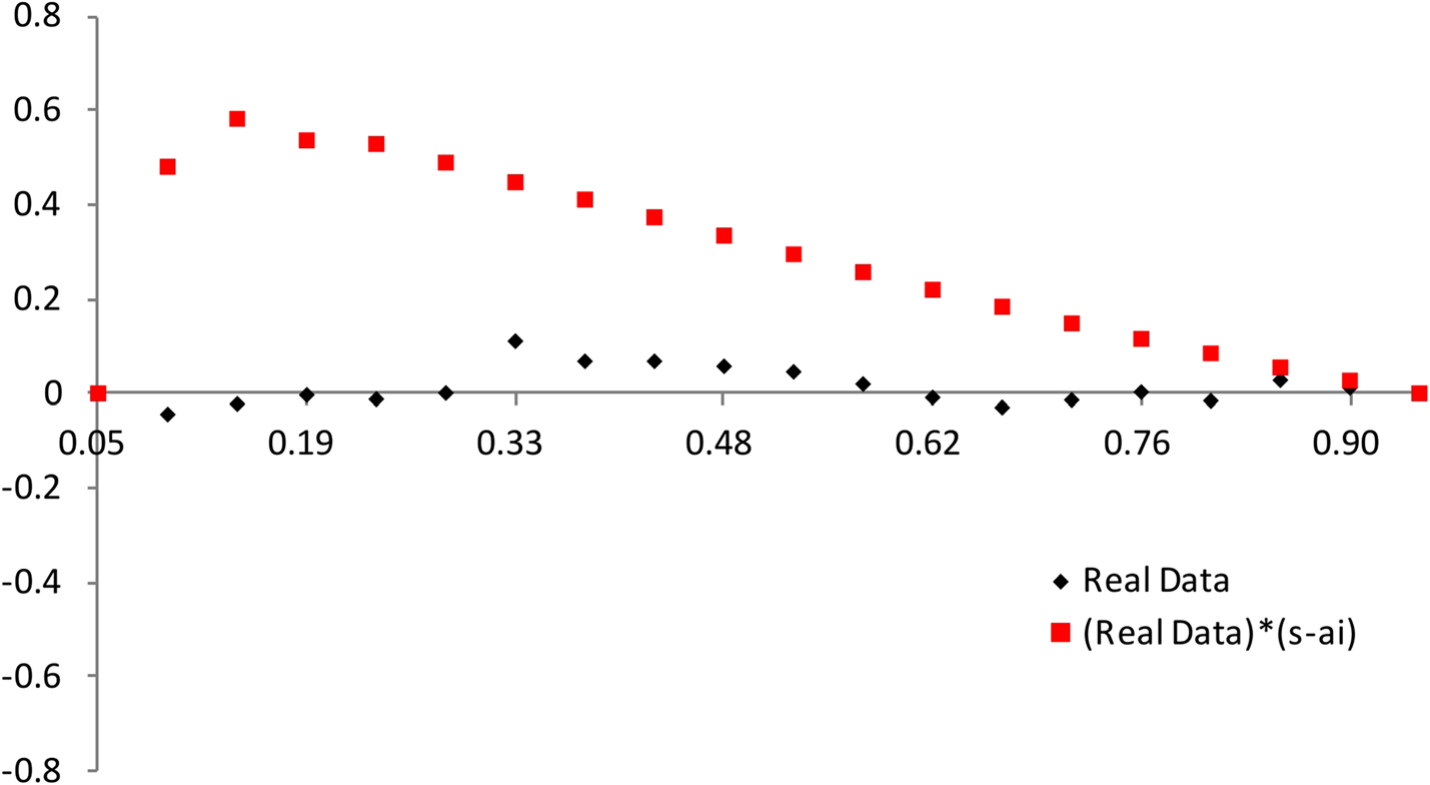
Plot based on the spacing of the EDF (example 2: complete data (*n* = 30))

**Fig. 12:**
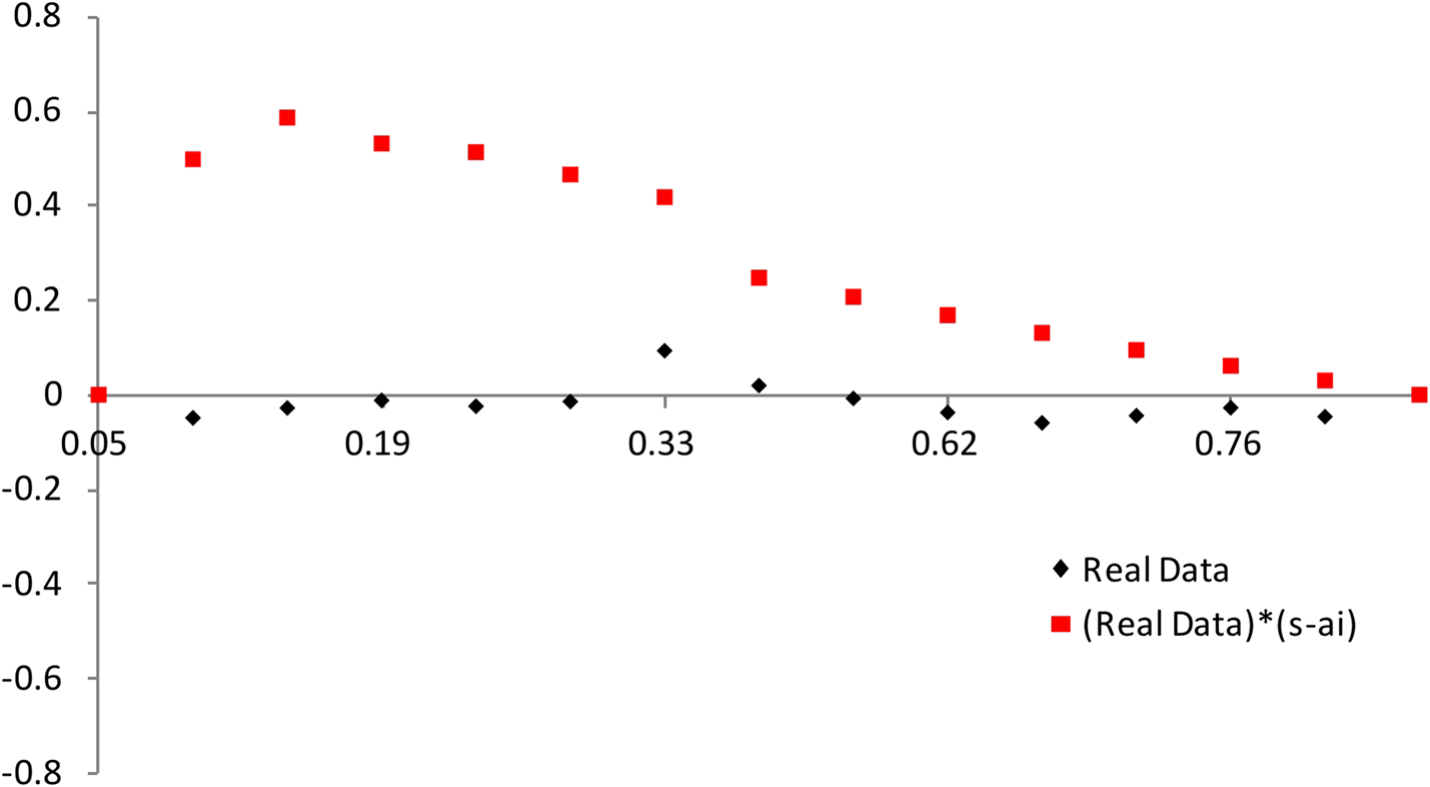
Plot based on the spacing of the EDF [example 2: multiply type-II censored data (*n* = 30, $a_j$ = 1, 5–13, 17–25, 28–30)]

We can picture the proposed plots for multiply Type-II censored samples using the AMLEs ${\hat{\lambda }}$ and ${\hat{\sigma }}$ (see Figs. 9, 10, 11, 12). It is easy to see that the plot based on spacing of EDF has good performance for complete data or multiply Type-II censored samples. The plot based on spacing of EDF is more sensitive than the modified NSLC plot.

## Conclusions

In most cases of censored and truncated samples, the maximum likelihood method does not provide explicit estimators. So we discussed another method for obtaining explicit estimators. We also proposed a simple graphical method for goodness on fit assessment based on multiply type-II censored samples using AMLEs.

We demonstrated that the proposed graphical method is a simple and fairly good approach for assessment of goodness of fit. We will need further study of the test statistics and the critical regions for testing distributions based on multiply type-II censored samples.
